# *In Vitro* Microleakage of Mineral Trioxide Aggregate, Calcium-Enriched Mixture Cement and Biodentine Intra-Orifice Barriers 

**DOI:** 10.22037/iej.2017.41

**Published:** 2017

**Authors:** Fatemeh Ramezanali, Sasan Aryanezhad, Fatemeh Mohammadian, Fatemeh Dibaji, Mohammad Javad Kharazifard

**Affiliations:** a *Department of Endodontics, Dental School, International Campus of Tehran University of Medical Sciences, Tehran, Iran; *; b * General Dentist, Tehran, Iran; *; c * Statistical Advisor, Dental Research Center, Tehran University of Medical Sciences, Tehran, Iran*

**Keywords:** Biodentine, Calcium-Enriched Mixture, Intra-Orifice Barrier, Microleakage, Mineral Trioxide Aggregate

## Abstract

**Introduction:**

This *in vitro* study compared the coronal microleakage of mineral trioxide aggregate (MTA), calcium-enriched mixture (CEM) cement and Biodentine as intra-orifice barriers.

**Methods and Materials:**

The study was conducted on 76 extracted single-canal human teeth. Their root canals were prepared using ProTaper rotary files and filled with gutta percha and AH-26 sealer using lateral condensation technique. Coronal 3 mm of the gutta percha was removed from the root canals and replaced randomly with MTA, CEM cement or Biodentine in the three experimental groups (*n*=22). A positive and a negative control group were also included (*n*=5). The entire root surfaces of all teeth were covered with two layers of nail varnish in such a way that only the access openings were not coated. In the negative control group, the access opening was also coated with nail varnish. All teeth were immersed in India ink and after clearing, the samples were evaluated under a stereomicroscope under ×10 magnification to assess the degree of dye penetration. The data were analyzed using the Kruskal-Wallis test. The level of significance was set at 0.05.

**Results:**

The negative control group showed no leakage while the positive control group showed significantly higher microleakage than the test groups (*P*>0.05). CEM cement had the lowest (0.175±0.068 mm) and MTA showed the highest dye penetration (0.238±0.159 mm) among the experimental groups; although these differences were not statistically significant (*P*=0.313).

**Conclusion:**

CEM cement exhibited the least microleakage as an intra-orifice barrier in endodontically treated teeth.

## Introduction

Bacteria and their byproducts are responsible for pulp necrosis and periapical diseases. Periapical health depends on elimination of microorganisms from the root canal system and prevention of their recolonization in the root canals [1]. Reentry of bacteria or their products into the root canals through the coronal opening or the apex, compromises the success of non-surgical endodontic treatment [2]. Evidence shows that secondary microleakage due to insufficient coronal seal is among the most important factors responsible for failure of root canal treatment [3]. Ray and Trope [4] showed that the quality of coronal restoration may be more important than the quality of root canal filling when it comes to periradicular health. Factors such as fracture of tooth structure, loss of temporary restorative materials, marginal leakage of final restoration and recurrent caries may be responsible for coronal microleakage [5]. 

To date, different materials and techniques have been suggested to decrease coronal microleakage. Placement of intra-orifice barriers is among the most efficient techniques in improvement of coronal seal in endodontically treated teeth. This technique includes application of sealing plugs into the root canal orifice immediately after removal of coronal gutta-percha and sealer [6]. 

Several materials such as Intermediate Restorative Material (IRM), amalgam, Cavit, glass ionomer cement, composite resin and Super-EBA have been used as intra-orifice barriers to prevent microleakage [7, 8]. Mineral trioxide aggregate has also been used for this purpose. Its chemical formulation resembles that of *type I* Portland cement and is a combination of dicalcium silicate, tricalcium silicate, tricalcium aluminate, tetracalcium aluminoferrite and bismuth oxide [9]. One important property of MTA is its ability to resist leakage due to its high marginal adaptation [10]. Due to these optimal properties, MTA is used not only as a root end filling material during periapical surgery but also as a suitable material for pulp capping, pulpotomy and perforation repair [11]. However, it has some drawbacks such as long setting time and difficult handling [12]. Some other materials were introduced to overcome the limitations of MTA such as calcium-enriched mixture (CEM) cement, which is composed of several calcium compounds including calcium oxide, calcium hydroxide, calcium carbonate, calcium silicate and calcium phosphate. When mixed with an aqueous-based solution, a bioactive material rich in calcium and phosphate is formed. In addition to clinical efficacies similar to that of MTA, CEM cement has easy handling and setting time of less than 1 h [13, 14]. 

Biodentine is supplied in the form of a powder in a capsule along with a liquid in a pipette. The powder contains tricalcium silicate, dicalcium silicate, calcium carbonate, calcium oxide, iron oxide and zirconium oxide. The liquid contains calcium chloride and water-soluble polymer. Biodentine is a suitable material for permanent restoration of dentin as well as endodontic purposes due to optimal properties such as remineralization of dentin, mechanical properties similar to those of dentin, easy use and handling, short setting time, resistance against leakage and being non-toxic [15, 16]. 

Limited studies are available on the efficacy of MTA and CEM cement as intra-orifice barriers. No study is available on Biodentine since it is a newly introduced material. Thus, considering the importance of coronal seal and the role of intra-orifice barrier in decreasing bacterial microleakage, the need for finding an affordable material with optimal properties for use as an intra-orifice barrier is clear. Thus, this *in vitro* study aimed to assess the coronal microleakage of MTA, CEM cement and Biodentine intra-orifice barriers.

## Materials and Methods

This *in vitro*, experimental study was conducted on 76 extracted human single-rooted, single canal teeth with no caries, root resorption or curvature. To ensure absence of cracks, the teeth were evaluated under a light stereomicroscope. The surface of all teeth was cleaned from tissue residues using a periodontal curette and the teeth were stored in 0.5% chloramine T solution until the experiment. To standardize root length, tooth crowns were cut by a high-speed handpiece under copious water irrigation to reach a standard root length of 14±0.5mm. Working length was measured using a #10 K-file (Dentsply Maillefer, Ballaigues, Switzerland). The file was inserted into the root canal until its tip was visible at the apex. Working length was determined 0.5 mm short of this length. Root canals were then prepared using ProTaper Universal (Dentsply Maillefer, Ballaigues, Switzerland) rotary files up to F3 using the crown-down technique. Between filings, the root canals were irrigated with 3 mL of 2.5% sodium hypochlorite. Finally, the root canals were rinsed with 5 mL of 17% EDTA (Prime Dental Products, Mumbai, India) for 1 min followed by 3 mL of 2.5% sodium hypochlorite and a final rinse with 5 mL of saline. The root canals were then dried with paper points and filled with gutta-percha (Aria Dent, Tehran, Iran) and AH-26 sealer (DeTrey, Konstanz, Germany) to the working length using the lateral condensation technique. 

Using a heat carrier, 3 mm of coronal gutta percha was removed and the remaining gutta-percha was vertically condensed. A periodontal probe was used to control the depth of intra-orifice cavity. The residual sealer on dentinal walls was removed using a cotton pellet dipped in alcohol. The teeth were numbered and assigned into three experimental (*n*=22) and two control (*n*=5) groups with simple random sampling. Coronal orifice of the test groups was filled with the sealing materials with 3 mm depth and MTA (Angelus, Londrina, Brazil), CEM cement (BioniqueDent, Tehran, Iran) and Biodentine (Septodont, Saint Maurdes Fosses, France) were used in groups 1, 2 and 3, respectively according to the manufacturers’ instructions. The samples were stored in 100% humidity at 37^°^C for 48 h. 

In all groups except for the negative control group, two layers of nail varnish were applied to the entire root surface except for the root canal orifice. In the positive control group, no sealing material was placed in the coronal orifice. In the negative control group, no intra-orifice barrier was placed either but the entire tooth surfaces (of the crown and the root) were coated with nail varnish. 

The samples were submerged in a vacuum flask containing India ink and were subjected to 75 Torr pressure under vacuum and remained in the dye for seven days. To eliminate the residual dye from the external surfaces, the samples were rinsed under running tap water and the nail varnish was completely removed from the surfaces as well. The following protocol was used for clearing: For demineralization, the samples were immersed in 5% nitric acid for five days, which was refreshed daily. The samples were rinsed under running water and stored in separate containers. For the purpose of dehydration, the teeth were immersed in 5 mL of ethyl alcohol with 80 and 90% concentrations for 12 and 2 h, respectively. To complete the process of clearing, the samples were stored in 6 mL of methyl salicylate solution. 

The experimental specimens were observed 360 degrees and leakage was measured in mm by a blind calibrated examiner to the greatest penetration under ×10 magnification of stereomicroscope (Zeiss, Munich, Germany) from the coronal extent of the orifice material. 

The mean microleakage measured for the test groups were statistically analyzed using the Kruskal-Wallis test. Statistically significant differences among the groups were set at *P*<0.05.

## Results

**Figure 1:**
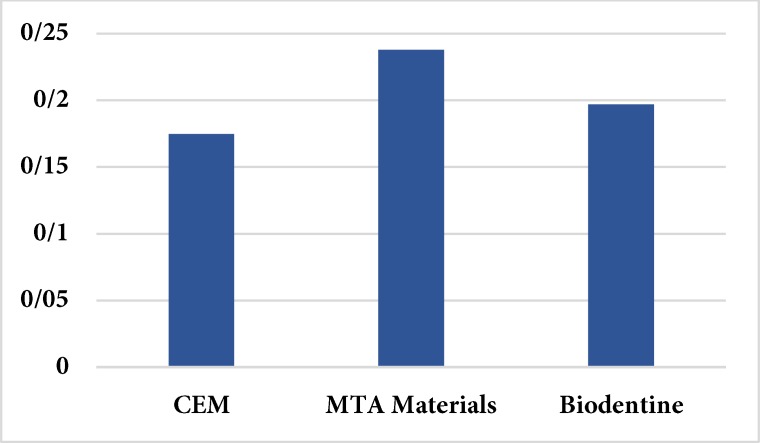
The mean dye microleakage of test groups; (CEM, calcium enriched mixture; MTA, mineral trioxide aggregate

In the positive control group, dye penetration was noted in the entire root canal length and a significant difference was noted in this regard between the positive control and other groups (*P*<0.05). Samples in the negative control group showed no evidence of dye penetration The lowest mean of dye penetration was found in CEM cement group (0.175±0.068 mm), followed by Biodentine (0.197±0.090 mm) and MTA (0.238±0.159 mm) (Figure 1). Kruskal-Wallis test indicated that the test groups did not differ significantly in dye penetration (*P*=0.313). 

## Discussion

Due to numerous excellent properties, MTA has extensive applications in endodontics [11, 12]. The setting expansion of MTA helps to provide an optimal seal and marginal adaptation. However, MTA has some drawbacks as well. Thus, search is ongoing for alternatives with more suitable properties [17]. 

Biodentine is a recently introduced dental material, which has not been evaluated for use as an intra-orifice barrier so far. Studies on the application of CEM cement for this purpose are scarce as well [18]. Thus, the current study aimed to assess the coronal microleakage of MTA, CEM cement and Biodentine when used as intra-orifice barriers. The results revealed no significant difference among the three materials in terms of microleakage. 

Conventional root canal filling materials such as gutta-percha and sealer have low resistance to microleakage. Thus, the coronal part of the root canal must be tightly sealed to prevent treatment failure. Intra-orifice barriers are often used for this purpose [19]. Wolcott *et al.* [20] discussed the required properties of an intra-orifice barrier. According to them, intra-orifice barriers must have easy application, bond to tooth structure, provide a tight seal against microleakage, should be distinguishable from the tooth structure and not interfere with the final restoration of the tooth [20]. Although previous studies have shown the optimal efficacy of intra-orifice barriers, no consensus has been reached on a specific protocol or material for use as a coronal barrier in endodontically treated teeth. Thus, attempts are ongoing to find a material with a potential to provide a long-term seal [20, 21]. 

Several methods have been used to assess the sealing ability and resistance to microleakage of materials used in endodontics such as dye penetration and extraction, fluid filtration, electrochemical methods, penetration of radioisotope tracers and use of bacterial leakage models [22]. These methods have their own advantages and limitations. Despite the limitations of dye leakage studies [23], this technique is among the most commonly used methods for this purpose due to its simplicity of use and low cost [24]. For this reason, we adopted the dye leakage technique in our study.

Dye penetration depends on several factors such as size of molecules, concentration of dye and the available surface area. In the current study, methylene blue dye was not used because it may be washed out during the process of clearing. Also, due to small size of its molecules, it is not suitable for microleakage assessment. We used India ink in the current study to assess microleakage because in contrast to methylene blue, India ink is stable during the experimental phases and does not stain dentin. It has no adverse effect on root canal sealers and only penetration of dye (and not the mixture of sealer and dye) is evaluated during the experiment [25, 26]. 

Our results are in line with those of Yavari *et al.* [18], who showed that CEM cement and MTA intra-orifice barriers were more effective than amalgam and composite resin for prevention of saliva leakage in endodontically treated teeth and no significant difference was found between them regarding the degree of leakage. Also according to Zarenejad *et al.* [27], MTA and CEM cement are considered as suitable intra-orifice barriers for providing coronal seal during walking bleaching. Some other studies on the sealing ability of CEM cement and MTA as root end filling materials showed that they both provided optimal coronal and apical seal [28-30]; this finding was in agreement with our results.

The results of studies comparing the sealing ability, microleakage and marginal adaptation of Biodentine and MTA have been controversial. For instance, some studies demonstrated that MTA had more favorable sealing ability and marginal adaptation than Biodentine when used as root-end filling materials or for furcal perforation repair [15, 31, 32]. In contrast, some other studies indicated that marginal adaptation and sealing ability of Biodentine were superior to those of MTA when used as root end filling material [33, 34]. In another study, no significant differences were found between bacterial leakage of MTA, CEM cement and Biodentine as furcation repair materials in primary molars [35]. Such a controversy in the results of studies may be due to differences in the understudy samples or different methodology of studies [31-35].

In general, it appears that all three materials tested in the current study are suitable for use as intra-orifice barriers in endodontically treated teeth since they have most of the ideal properties named by Wolcott *et al.* [20], for a coronal barrier such as providing excellent seal against microleakage and easy application. In conclusion, immediate placement of a suitable intra-orifice barriers such as CEM cement, Biodentine or MTA, prior to final restoration of tooth can effectively decrease the coronal microleakage and re-contamination of root canal contents.

## Conclusion

This study was conducted using dye penetration and the results showed that there were no differences regarding the coronal microleakage of experimental groups. However, CEM cement exhibited the least microleakage. It seems that CEM cement, Biodentine and MTA, are effective for providing an efficient coronal seal when used as an intra-orifice barriers in endodontically treated teeth. 
